# Pharmaceutical Systems as a Strategy to Enhance the Stability of Oxytetracycline Hydrochloride Polymorphs in Solution

**DOI:** 10.3390/pharmaceutics15010192

**Published:** 2023-01-05

**Authors:** Maria S. Bueno, Marcela R. Longhi, Claudia Garnero

**Affiliations:** 1 Departamento de Ciencias Farmacéuticas, Facultad de Ciencias Químicas, Universidad Nacional de Córdoba, Ciudad Universitaria, Haya de la Torre and Medina Allende, Science Building 2, Córdoba X5000HUA, Argentina; 2 Unidad de Investigación y Desarrollo en Tecnología Farmacéutica, CONICET, Consejo Nacional de Investigaciones Científicas y Técnicas, UNITEFA, Córdoba X5000HUA, Argentina

**Keywords:** oxytetracycline hydrochloride, cyclodextrin, amino acids, stability, NMR

## Abstract

In order to improve the stability of oxytetracycline hydrochloride, a polymorphic antibiotic set of novel binary systems were developed using β-cyclodextrin and amino acids with different acid-basic characteristics as ligands. The formation constants for each system containing β-cyclodextrin, L-aspartic acid, histidine and N-acetylcysteine were determined by Scott’s method and statistical studies. The structure of the binary systems with β-cyclodextrin and N-acetylcysteine was elucidated by NMR experiments. The effect β-cyclodextrin and N-acetylcysteine on the polymorph’s chemical stability in aqueous and phosphate buffered saline solutions at 25 °C was monitored by an optimized and validated high-performance liquid chromatography method. The combination of N-acetylcysteine with the three polymorphs and the β-cyclodextrin system obtained with the form III demonstrated a reduction in the degradation rate of oxytetracycline hydrochloride in the aqueous solution when compared to each free form, with an increase of 20 h in the half time. It evidences that the use of amino acids as ligands constitutes an interesting alternative for pharmaceutical areas. In conclusion, based on the results obtained, these pharmaceutical systems could be candidates for the development of a pharmaceutical formulation for the administration of the drug through reconstituted solutions using the binary system as a promising tool for improving the stability of oxytetracycline hydrochloride polymorphs in solution.

## 1. Introduction

Stability is a crucial factor of any active pharmaceutical ingredient (API) that must be considered during several stages as early formulation, development, production, storage, and commercialization. Chemical and physical stabilities have a significant impact on the safety and efficacy of API and drug products, which should be physically and chemically stable throughout their shelf life without being affected by stimuli such as temperature, moisture, and light. Knowing the impact of these environmental factors aids in the development of suitable manufacturing and storage conditions, retesting intervals, and the determination of the shelf life of an API [1,2,3]. Stability studies are important in the pharmaceutical industry, since they are required to satisfy regulatory agencies licensing requirements. These studies must be performed in accordance with the International Council of Harmonization (ICH) [4] or other regulatory guidelines [5,6,7].

Considering that drug degradation occurs in several stages via different pathways generating degradation products with adverse bioactivity and increased toxicity, the pharmaceutical field focused efforts in expanding numerous strategies for improving the API stability. Several studies have emerged in this context, reporting the potential use of cyclodextrins (CDs) for improving the stability through the formation of inclusion complexes, which encapsulates labile moieties of the drug molecules [8,9,10,11]. Among the CDs, β-cyclodextrin (β-CD) is widely used due to its numerous advantages, including a suitable cavity size for the inclusion of guest molecules, its non-toxicity, its biodegradability, and its reasonable price [12,13,14]. Recently, the stabilization of hydrochlorothiazide through incorporating it into β-CD cavity was reported. β-CD prevented the hydrolytic degradation of hydrochlorothiazide, which increased its half-life in aqueous solutions by nearly 28-fold [15]. Additionally, in the presence of β-CD the posaconazole stability was increased under oxidative stress, which was evidenced by an increment in half-life and shelf-life of the drug [16]. However, the effect of β-CD complexation on the stability of the liquid suspension cefixime dosage form was dependent on the complex preparation method, according to Pamudji et al. It was observed that the complex formed by freeze drying degraded faster than the complex produced by kneading [17].

Additionally, the interest in amino acids has grown recently. They are water-soluble carriers of relatively low molecular weights that have suitable structural characteristics (α-carboxylate, α-amino group, and side chains) for forming bonds with APIs. Numerous studies have demonstrated how amino acids can enhance the stability and safety of APIs [18,19,20,21,22,23].

**Figure 1:**
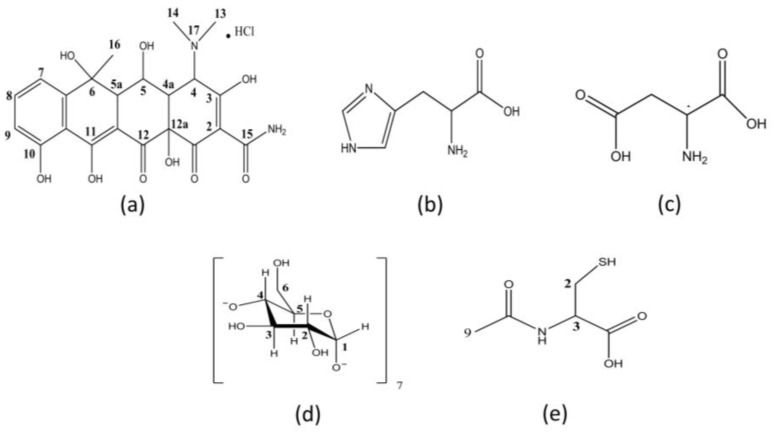
Molecular structure and NMR signal notation of (**a**) Oxytetracycline hydrochloride, (**b**) Histidine, (**c**) L-aspartic acid, (**d**) β-cyclodextrin, and (**e**) N-acetylcysteine.

Oxytetracycline hydrochloride (OxyCl) (Figure 1) is a polymorphic tetracycline antibiotic of the broad-spectrum that is frequently used for treating respiratory tract infections, urethritis, severe acne, and also in the treatment of multidrug resistant malaria. Recent studies evidence that OxyCl polymorphs exhibited similar antimicrobial activity [24]. OxyCl is known to undergo several chemical reactions. Due to its special macromolecular zwitterionic structure, it can generate three main degradation products under specific conditions such as acid or alkaline solution. The main degradation pathways include photodegradation, oxidative degradation, and hydrolysis, with the latter being influenced in an aqueous medium by factors such as temperature, pH, and light [25,26]. However, detailed information about the stability of each OxyCl polymorph cannot be found in the literature, therefore, it must be thoroughly investigated.

In this study, the objective was to determine the stability parameters of the OxyCl polymorphs (OxyCl-I, OxyCl-II, and OxyCl-III), analyzing in particular its degradation in aqueous and phosphate buffered saline (PBS) solutions, as well as the influence of novel binary systems developed to improve its stability in solutions. A detailed evaluation of several ligands (Figure 1) was performed in order to select those with the most effective interaction. Then, the association constant was assessed using spectroscopic measurements. After that, chemical stability studies were performed for the binary systems obtained with β-CD and N-acetylcysteine (NAC). To accomplish this, the samples in the solutions were analyzed using an optimized and validated high performance liquid chromatographic (HPLC) procedure. Furthermore, the interactions in these binary systems were investigated in detail using nuclear magnetic resonance (NMR) experiments.

## 2. Materials and Methods

### 2.1. Chemicals and Equipment

Oxytetracycline hydrochloride (OxyCl-I) was purchased from Todo Droga (Córdoba, Argentina). β-cyclodextrin (β-CD, MW = 1135) was kindly provided by Ferromet (agent in Argentina of Roquette (France)). L-aspartic acid (ASP) was provided by Anedra (Buenos Aires, Argentina), N-acetylcysteine (NAC) and Histidine (HIS) were purchased from Sigma-Aldrich (Milwaukee, WI, USA). All chemicals were of an analytical grade. Isopropyl alcohol and phosphoric acid were provided by Cicarelli (Santa Fe, Argentina). Ethanol was purchased from Sintorgan (Buenos Aires, Argentina). Acetonitrile J.T.Baker was supplied by Avantor Performance Materials (Xalostoc, México). Deuterium oxide (D_2_O) was provided by Sigma-Aldrich (Saint Louis, MO, USA).

A spectrophotometer, Cary 60 UV-Vis (Agilent Technologies, Santa Clara, CA, USA), with 10 mm of quartz cells was used for all spectral measurements.

The HPLC system consisted of an Agilent 1100 series pump, an autosampler, a multiple-wavelength ultraviolet-visible (UV-vis) detector, and Chemstation software Rev. B 04.03 (Agilent Technologies, Waldbronn, Germany).

### 2.2. Obtaining the Solid Forms of Oxytetracycline Hydrochloride

OxyCl solid forms were obtained as stated in our prior report [24]. OxyCl-II was produced by the slow evaporation of a saturated solution of commercial form (OxyCl-I) in isopropyl alcohol. OxyCl-III was obtained from a saturated OxyCl-I solution in ethanol, which was maintained at 8 °C for a week.

### 2.3. Preparation of Binary Systems

The binary systems of OxyCl polymorphs I, II, and III were prepared in a 1:1 molar ratio with the ligands β-CD (OxyCl:β-CD), ASP (OxyCl:ASP), NAC (OxyCl:NAC), and HIS (OxyCl:HIS). An adequate amount of each polymorph was dissolved in water, then a solution of each ligand was added in order to obtain the equimolar ratio.

### 2.4. Determination of Association Constants from UV-Visible Spectroscopy

UV-vis data were used to calculate the association or binding constants (*K*_a_) of OxyCl polymorphs with each ligand in an aqueous solution by different treatment analyses. The concentration of OxyCl was kept constant (0.068 mM), while the concentration of ligands increased from 0.068 to 10 mM.

#### 2.4.1. Scott’s Plot Method

*K*_a_ values were determined by applying the formula of Benesi–Hildebrandt, adjusted by Scott [27] utilized for a complex of 1:1 stoichiometry:

(1) $$\frac{\left[L\right]}{\Delta A_{obs}}=\frac{\left[L\right]}{\Delta A_{c}}+\frac{1}{K_{a}\times \Delta A_{c}\;}$$

where [*L*] is the molar concentration of the ligand, ∆*A_obs_* is the variation in the absorbance observed for the OxyCl solid form for a given [*L*], Δ*A_c_* is the variation in the absorbance between the complex and the free solid form.

#### 2.4.2. Statistical Treatment of Data

Another model for measuring binding constants in data analysis utilizes least-squares analysis. The general approach is to use the linear regression method with a model matrix to find the binding association constant and then apply least-squares procedures to a linear transformation. The normal equations can be written in matrix form as:

(2) M×P=Q

where

(3) $$M=\left[\begin{array}{cc} n & \sum x\\ \sum x & \sum x^{2} \end{array}\right]$$

(4) $$P=\left[\frac{a}{b}\right]$$

(5) $$Q=\left[\frac{\sum y}{\sum xy}\right]$$

then:

(6) $$K_{a}=\frac{a}{b}$$

### 2.5. Stability Study Design

#### 2.5.1. Chemical Stability Study

The kinetics of degradation of OxyCl polymorphs both in the presence and absence of ligands were investigated in the aqueous solutions and PBS.

Stock solutions of each solid form, as well as each ligand β-CD, ASP, and NAC, were prepared. These stock solutions were diluted to a final concentration of 30 µg/mL in water and PBS solutions to prepare the test solutions. The solutions containing ligands were prepared in the molar ratio of 1:1. These solutions were stored with light protection in order to diminish any photolytic effects in a water bath at a constant temperature of 25.0 ± 0.1 °C. Samples were withdrawn at appropriate time intervals and immediately analyzed by HPLC. The observed pseudo first-order rate constants (*k*_obs_) for the OxyCl polymorphs degradation were obtained from a linear regression analysis of the natural logarithm of the remaining solid forms plotted against time.

#### 2.5.2. Chromatographic Conditions

An HPLC-UV procedure under isocratic conditions [28] was optimized. The results were expressed as the mean of the three determinations of samples prepared in duplicate. The HPLC system was an Agilent 1100 (Agilent Technologies, Waldbronn, Germany) equipped with a column Gemini C18 250 mm × 4.6 mm i.d. filled with 5 μm particles and a precolumn (guard cartridge SecurityGuard C18 4 mm × 3.0 mm i.d.) supplied by Phenomenex (Phenomenex Inc., Torrance, CA, USA). To avoid interference from degradation products, the UV detection was performed at a wavelength of 355 nm. The mobile phase was acetonitrile-water adjusted to a pH of 3 with phosphoric acid (20:80, *v/v*), and a flow rate of 1 mL/min was used. The column temperature was 25 °C and the injection volume was 50 µL.

#### 2.5.3. Validation of Chromatographic Method

This HPLC method was validated in accordance with standard guidelines [29]. Linearity and range were evaluated by constructing calibration curves in triplicates at seven concentration levels. The detection (LOD) and quantification (LOQ) limits were determined by using the standard deviation (SD) and the slope of the calibration graphs. Precision and accuracy were established by testing three different OxyCl concentrations in triplicate covering the range. Precision was analyzed by calculating the percent relative standard deviation (% R.S.D.) of tests performed on the same day (intra-day) and on different days (inter-day). The accuracy was reported as the percent recovery (% recovery) determined by the assay.

### 2.6. Nuclear Magnetic Resonance Spectroscopy Study

NMR studies were used to investigate the interaction between the OxyCl solid forms and the ligands. The proton nuclear magnetic resonance (^1^H NMR) and two-dimensional nuclear Overhauser effect spectroscopy (2D-NOESY) experiments were performed on a Bruker Avance II High Resolution Spectrometer, at 298 K using 5 mm sample tubes. The spectra were obtained at 400.16 MHz. The solutions of pure compounds and their equimolar ratio associations were prepared in D_2_O at a concentration of 15 mM. The chemical shifts (*δ*) were reported as ppm, and were referred to the residual solvent signal (4.80 ppm). Induced changes in the *δ*, originated by the interaction with the ligands, were calculated as:

(7) Δ*δ* = *δ*_system_ − *δ*_free_

The 2D-NOESY spectra of the binary system were recorded 1D-NOESY using selective refocusing with a shaped pulse; dipolar coupling may be due to NOE or chemical exchange. The mixing time for NOESY was 0.3 s. The spectra were acquired with f1 channel-90 degree high power pulse (9.06 ms); the relaxation delay was 1 s.

The NMR data were processed with the MestReNova software (version 6.0.2-5475).

## 3. Results and Discussion

### 3.1. Determination of the Apparent Binding Constants

The absorption spectra of the OxyCl:β-CD, OxyCl:ASP, OxyCl:HIS, and OxyCl:NAC for the three solid forms have been measured in order to investigate the molecular interactions focused on the selection of optimum ligand.

**Figure 2:**
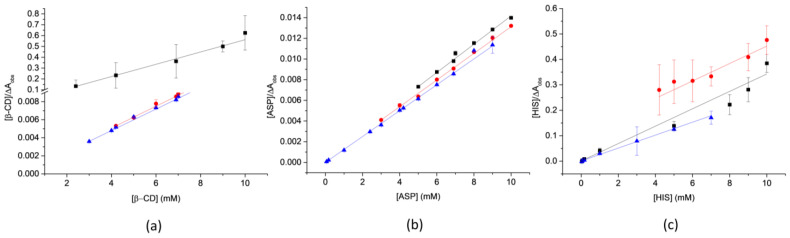
Scott plot of systems containing: (**a**) β-CD; (**b**) ASP; (**c**) HIS; (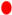) OxyCl-I, (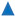) OxyCl-II, and (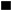) OxyCl-III.

The association constant (*K_a_*) for the formation of binary systems was estimated from changes in the intensity of the absorption at 355 nm with increasing ligand concentration using double reciprocal plots of Scott´s equation. As seen in Figure 2, the systems containing the ligands β-CD, ASP, and HIS have a linear correlation, confirming its adjustment for Scott’s model. However, deviations in straight lines were detected for the binary systems with NAC, which determined it was impossible to calculate an association constant assuming this model. To determine the *K_a_* for OxyCl:NAC systems, a model matrix that applied least-squares techniques for the linear transformation of the data was used.

Table 1 are summarized the values of *K*_a_ for the binary systems. Different interactions had been observed between each OxyCl polymorph and the ligands ASP, β-CD, HIS, and NAC. According to these results, the most stable systems were OxyCl-I:ASP, which exhibited a constant of 34,645 M^−1^, and OxyCl-II: β-CD with a *K*_a_ of 15,425 M^−1^. Nevertheless, HIS had a higher affinity for OxyCl-III, whereas OxyCl-I and OxyCl-II had weaker interactions. As a result, it was decided that HIS should not be studied further.

### 3.2. Stability Studies

#### 3.2.1. Validation of Chromatographic Method

An efficient, precise, and simple reversed-phase HPLC (RP-HPLC) procedure was adapted from the bibliography [28].

The specificity parameter was initially validated in order to evaluate potential interferences from degradation products. The OxyCl samples were previously subjected to stress conditions for this purpose. For 1 h, acidic and basic solutions containing a mixture of 10 mg powder with 20 mL of 1 M HCl or NaOH solution, respectively, were refluxed with heating and light protection. The wavelength of maximum absorption of the degradation products differed from that of OxyCl, being 250 nm and 355 nm, respectively. It confirmed that the method was specific, and OxyCl could then be determined without interference from the degradation products (see Supplementary Materials, Figure S1). Moreover, in terms of peak shape and its retention time, the chromatograms obtained for aqueous solutions in the presence of the ligands ASP, β-CD, and NAC (see in Supplementary Material Figures S2–S4, respectively) were similar to those obtained in the absence of the ligands under the same experimental conditions, indicating that the molecular interactions in the binary systems had no interference for the drug determination.

Subsequently, the parameters of linearity and range, limit of detection (LOD), limit of quantitation (LOQ), precision, and accuracy were also examined (Table 2). The correlation between the peak area ratio and OxyCl concentration was linear over the range. Recovery studies were used to evaluate precision and accuracy at three concentration levels (Table 3). The results of experiments revealed %R.S.D. values lower than 2%, which were considered satisfactory and demonstrated the precision of the method. The precision test performed on the same day demonstrated the repeatability of the method, whereas the intermediate precision was determined on different days. Furthermore, optimal recovery values for OxyCl (within 100 ± 2%) were obtained at each concentration, indicating the accuracy of the method.

#### 3.2.2. Degradation Studies

The validated stability indicating the RP-HPLC method was applied for the analysis of the kinetics of the degradation process of each OxyCl solid form.

It is important to mention that the results presented in these studies represent the first report about OxyCl polymorphs stability. The degradation curves of each OxyCl polymorph and its binary systems with β-CD, NAC, and ASP in an aqueous solution, under identical experimental conditions, were compared. It showed linear relationships, indicating that the chemical degradation in an aqueous solution followed an apparent first-order kinetics. The kinetic parameters, which are important in the pharmaceutical sector, were determined using a first-order reaction model that could be expressed by the following equation:

ln C_t_ = C_0_ − k t (8)

where C_t_ and C_0_ were the concentrations of OxyCl at different reaction times and at the initial time, respectively, k was the degradation rate constant, and t was time. As a result, the slope of the fitted lines could be used to determine k.

Furthermore, linear regression analysis was used to estimate the intrinsic rate constant of degradation of the free OxyCl polymorphs (k_0_), the observed rate constant of OxyCl polymorphs in the presence of ligands (k_obs_), the half-life (t_50_), and the time of 10% degradation of the drug (t_90_).

The kinetic parameters obtained for the degradation reactions in aqueous solutions (Table 4) demonstrated that OxyCl-II and OxyCl-III degraded faster than OxyCl-I, with a degradation rate of OxyCl-I < OxyCl-III < OxyCl-II. Interestingly, the ligands studied showed different effects on OxyCl polymorphs stability. The relation k_0_/k_obs_ showed the destabilizing effects for all binary systems produced using ASP as the ligand, which could be attributed to the acidification of media (around pH 4) by ASP. Conversely, the binary systems obtained with β-CD only showed stabilizing effects for OxyCl-III polymorph, evidencing clear differences in the interaction between the ligand and each polymorph. Particularly, the binary systems containing NAC demonstrated a stabilizing effect with the slowest degradation, as compared to the degradation of free solid forms as evidenced by the relation k_0_/k_obs_. It could be explained by assuming that the interaction with NAC reduced the reactivity of hydrolysis in aqueous media.

According to these results, it was interesting to investigate the stability of OxyCl polymorphs and its binary systems with NAC in a simulated physiological medium by using PBS. The degradation plots were linear, indicating apparent first-order kinetics. The results summarized in Table 5 showed that OxyCl polymorphs had a higher stability in the PBS solution (pH 7.4) than in an aqueous solution (around pH 4), which could attribute to the pH PBS solution resulting as less favorable for the hydrolytic degradation of OxyCl. It was observed that OxyCl-I degraded at a faster rate than OxyCl-II and OxyCl-III, with a degradation rate of OxyCl-II < OxyCl-III < OxyCl-I. 

Surprisingly, the studied binary systems exhibited a rate of degradation considerably higher than the free polymorphs in PBS solution (Table 5); as demonstrated by the analysis of the relation k_0_/k_obs_. Moreover, the results obtained clearly showed that the degradation of OxyCl:NAC systems in aqueous solutions was much slower than in PBS solutions.

From these values, a different interaction in the two media between the drug and the NAC could be deduced, possibly due to the buffer effects. As was previously reported, an inhibition of the interaction between OxyCl and NAC caused by salt formation with the components of the buffer [30] could explain the catalysis of the degradation. Furthermore, the fact that the pH of OxyCl:NAC solutions in PBS was about 4 suggested that the destabilizing action on OxyCl polymorphs might be a catalytic combined degradation when considering the acidification media caused by NAC.

### 3.3. Nuclear Magnetic Resonance Spectroscopy

^1^H NMR experiments were conducted in order to study and distinguish the structures of the three solid forms of OxyCl, and to investigate the interaction between the OxyCl-I, OxyCl-II, and OxyCl-III polymorphs and the ligands β-CD and NAC, which demonstrated a stabilizing effect in the solution. Table 6 summarizes the proton signals assignment for each OxyCl polymorph and the ligands. The individual protons were assigned based on their position in the chemical structures, as shown in Figure 1, in accordance with previous reports [31,32,33].

The ^1^H NMR spectra of OxyCl-II and OxyCl-III polymorphs revealed variations in the chemical shifts, with respect to OxyCl-I (see Figure S5 in Supplementary Material). In particular, OxyCl-II exhibited changes in the chemical shifts of the signals for protons H4, H4a, H5, and H5a, the aromatic protons (H6, H7, and H8) and the protons of the amide group. OxyCl-III exhibited differences in the signals corresponding to the amide group protons, H5, and the aromatic protons (H6, H7, and H8). Therefore, it can be seen that the ^1^H NMR spectra identified polymorphic OxyCl forms.

Changes in the signals of the ^1^H NMR spectra corresponding to binary systems when compared to individual component spectra evidenced interactions between the molecules, as can be seen in Table 7. Due to the destabilizing effect observed in the stability studies, ASP was excluded from these experiments. The spectra of the binary systems with β-CD showed major displacements in the chemical shifts of β-CD protons located inside the cavity (H3 and H5) with shielding effects, while only minor changes were observed for the protons located outside it (H1, H2, H4, and H6). The changes observed for H3 were greater than for H5 protons, indicating a partial insertion of the OxyCl molecule into the β-CD cavity from the wide side, due to the fact that H3 protons were near the wide side of the cavity as shown in the literature for complexes as alpinetin:hydroxypropyl-β-CD [34], rosuvastatin calcium:methyl-β-CD [35], and sulfasalazine-β-CD [36].

When the signals of OxyCl polymorphs were analyzed, a deshielding effect for the H4, H4a, and H5a protons was observed, which could be attributed to an alteration in the local polarity of these protons, while H13 and H14 showed insignificant displacement. It was suggested that the –OH group linked to C5 of the OxyCl molecules interacted with the macromolecule cavity, probably by hydrogen bond interactions.

The spectra of OxyCl:NAC systems showed significant displacements in the chemical shifts of H4, H4a, and H5a protons of the drug. Moreover, a deshielding effect of the H3 NAC proton was observed that could be attributed to the hydrogen bond interactions. This behavior was similar to that reported by Aiassa et al. [37], who detailed hydrogen bond interactions between the carbonyl and amine groups of NAC and carbonyl groups of chloramphenicol.

**Figure 3:**
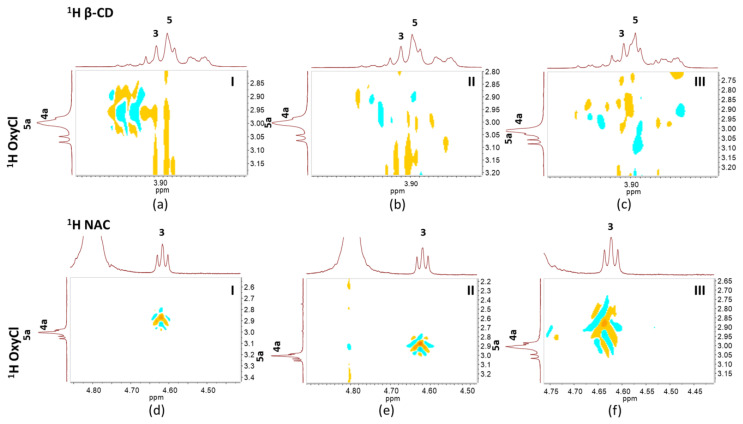
Partial contour plot of the 2D NOESY spectrum showing the intermolecular proximities of (**a**) OxyCl-I:β-CD, (**b**) OxyCl-II:β-CD, (**c**) OxyCl-III:β-CD, (**d**) OxyCl-I:NAC, (**e**) OxyCl-II:NAC, and (**f**) OxyCl-III:NAC.

To explore the intermolecular interactions in these binary systems, 2D NOESY spectra were analyzed (Figure 3). It was possible to observe that the three solid forms of OxyCl interacted similarly with each ligand (β-CD and NAC). The 2D NMR spectra of OxyCl:β-CD systems showed several cross peaks of H3 and H5 protons of β-CD with H4, H4a, and H5a protons of OxyCl, whereas no cross peak was detected between aromatic ring protons (H7, H8, and H9) of OxyCl and protons within the β-CD, thus confirming the partial inclusion of OxyCl polymorphs in the β-CD cavity. It showed how several tetracycline drugs and β-CD interacted differently. The literature, for instance, described ROESY experiments that demonstrated the inclusion of doxycycline in the β-CD cavity via its aromatic side [27]. Whereas, the 2D NMR spectra of the OxyCl:NAC systems showed an appreciable correlation (spatial proximity) of the H3 NAC proton with the H4, H4a, and H5a OxyCl protons, demonstrating the NAC interaction with –OH group linked to C5 of OxyCl polymorphs.

## 4. Conclusions

These studies have demonstrated that the ligands β-CD, ASP, and NAC are capable of interacting with OxyCl polymorphs producing binary systems. NMR studies confirmed the formation of the OxyCl:β-CD and OxyCl:NAC binary systems in solutions, showing that the OH bound to the C5 was involved in the interactions with the ligand. Furthermore, these systems showed different effects on the chemical stability of the drug. For stability studies, a chromatography stability-indicating method was validated, which was successfully applied to the measurement of OxyCl samples in a solution subjected to the kinetic degradation studies, with no interference from its degradation products. Differences were found in the stability of OxyCl polymorphs. Moreover, the OxyCl-I:NAC, OxyCl-III:β-CD, and OxyCl-II:NAC systems showed the greatest improvement in chemical stability of the polymorphs in an aqueous solution, with degradation rates always lower than that of the free molecule.

These binary systems are promising alternatives for the development of pharmaceutical formulations for drug administration via reconstituted solutions with improved biopharmaceutical properties in aqueous solutions, considering that variations in the stability of OxyCl polymorphs could result to variations in the drug bioavailability. Additionally, its potential use in drug repositioning is considered a promising strategy with applications against a variety of therapies.

## Figures and Tables

**Table 1:** Apparent binding constants *K*_a_ (M^−1^).

Polymorph	Ligands			
	β-CD	ASP	HIS	NAC
OxyCl-I	3761	34,645	224	2042
OxyCl-II	15,425	3063	323	4582
OxyCl-III	3607	1771	8247	3897

**Table 2:** Validation report of HPLC method for determination of OxyCl.

Parameters	Results	
**Linearity**	Regression equation ^a^	Y = 14.544 X − 2.603
	Correlation coefficient (r^2^)	0.994
	Linear range (μg/mL)	5.0–40.0
**Detection limit**	0.38 (μg/mL)	
**Quantification limit**	1.15 (μg/mL)	

^a^ X is the concentration of OxyCl in μg/mL; Y is the peak area at 355 nm.

**Table 3:** Validation parameters of HPLC method for determination of OxyCl.

Nominal Concentration (μg/mL)	Measured Concentration (μg/mL)	Accuracy (% Recovery)	Precision (% R.S.D.)
**Intra-day (repeatability)**			
**20**	20.1 ± 0.2	100.36	1.26
**25.2**	25.2 ± 0.4	100.22	1.73
**30.2**	30.2 ± 0.1	99.86	0.32
**Inter-day (intermediate precision)**			
**20.2**	20.5 ± 0.1	101.38	0.55
**25.5**	26.0 ± 0.3	101.93	1.33
**29.9**	30.5 ± 0.1	101.96	0.42

**Table 4:** Kinetic parameters of the degradation process in aqueous solution at 25.0 ± 0.1 °C.

Solids	k_0_ (h^−1^)	k_obs_ (h^−1^)	t_50_ (h)	t_90_ (h)	k_0_/k_obs_
OxyCl-I	0.0102		68	10	
OxyCl-I:β-CD		0.0134	52	8	0.76
OxyCl-I:ASP		0.0143	48	7	0.71
OxyCl-I: NAC		0.0093	74	11	1.10
OxyCl-II	0.0121		57	9	
OxyCl-II:β-CD		0.0132	53	8	0.92
OxyCl-II:ASP		0.0142	49	7	0.85
OxyCl-II:NAC		0.0079	73	11	1.53
OxyCl-III	0.0116		60	9	
OxyCl-III:β-CD		0.0087	80	12	1.33
OxyCl-III:ASP		0.0300	23	4	0.39
OxyCl-III:NAC		0.0095	87	13	1.22

**Table 5:** Kinetic parameters of the degradation process in PBS at 25.0 ± 0.1 °C.

**Solids**	**k_0_ (h^−1^)**	**k_obs_ (h^−1^)**	**t_50_ (h)**	**t_90_ (h)**	**k_0_/k_obs_**
OxyCl-I	0.009		81	12	
OxyCl-I:NAC		0.012	58	9	0.75
OxyCl-II	0.005		139	21	
OxyCl-II:NAC		0.022	32	5	0.22
OxyCl-III	0.007		102	15	
OxyCl-III:NAC		0.013	53	8	0.54

**Table 6:** ^1^H NMR chemical shifts (*δ*) of OxyCl forms, β-CD, and NAC.

*δ* (ppm)						
Assignment	Polymorph			Assignment	Ligand	
	OxyCl-I	OxyCl-II	OxyCl-III		NAC	β-CD
**H4**	4.167	4.280	4.167	**H2**	2.992	
**H13-14**	3.054	3.054	3.061	**H3**	4.569	
**H4a**	2.843	2.939	2.843	**H9**	2.099	
**H5**	3.961	3.949	3.964			
**H5a**	2.927	2.968	2.927	**H1**		5.068
**H16**	1.814	1.803	1.821	**H2**		3.647
**H7**	7.254	7.246	7.261	**H3**		3.965
**H8**	7.624	7.617	7.631	**H4**		3.582
**H9**	7.037	7.026	7.044	**H5**		3.870
**NH2**	8.352	8.254	8.360	**H6**		3.908

**Table 7:** ^1^H NMR chemical changes (∆*δ*) in the binary systems.

Δ*δ* = *δ*_system_ − *δ*_free_						
Assignment	OxyCl-I:β-CD	OxyCl-II:β-CD	OxyCl-III:β-CD	OxyCl-I:NAC	OxyCl-II:NAC	OxyCl-III:NAC
**H4**	0.152	0.044	0.179	0.172	0.06	0.178
**H13-14**	0.005	0.009	0.009	−0.007	−0.004	0.002
**H4a**	0.095	0.045	0.187	0.143	0.049	0.147
**H5**	0.015	0.032	0.016	−0.007	0.008	0.011
**H5a**	0.071	0.035	0.085	0.076	0.037	0.078
**H16**	0.001	0.046	0.027	−0.005	0.008	0.007
**H7**	0.008	0.021	0.009	−0.005	0.005	0.005
**H8**	0.011	0.022	0.012	−0.007	0.003	0.005
**H9**	0.004	0.018	0.005	−0.006	−0.003	0.014
**Ligands**						
	**β-CD**					
**H1**	0.002	0.007	0.007			
**H2**	0.009	0.014	0.019			
**H3**	−0.05	−0.045	−0.04			
**H4**	0.001	0.006	0.006			
**H5**	−0.036	−0.024	−0.027			
**H6**	0.007	0.012	0.005			
				**NAC**		
**H2**				−0.012	−0.006	−0.016
**H3**				0.047	0.05	0.054
**H9**				−0.012	−0.009	−0.009

## Data Availability

Not applicable.

## Supplementary Materials

The following supporting information can be downloaded at: https://www.mdpi.com/article/10.3390/pharmaceutics15010192/s1, Figure S1: Chromatograms of OxyCl polymorphs and degradation products obtained from acid and basic reactions; Figure S2: Chromatograms of binary systems with ASP in aqueous solution; Figure S3: Chromatograms of binary systems with β-CD in aqueous solution; Figure S4: Chromatograms of binary systems with NAC in aqueous solution; Figure S5: ^1^H-NMR chemical assignment of (a) OxyCl-I, (b) OxyCl-II and (c) OxyCl-III.
