# Supplementary material for: Pharmaceutical Systems as a Strategy to Enhance the Stability of Oxytetracycline Hydrochloride Polymorphs in Solution
Source: Pharmaceutics. 2023 Jan 5;15(1):192. doi: 10.3390/pharmaceutics15010192 (PMC9862800; doi:10.3390/pharmaceutics15010192)
Supplement: Supplementary file 1 [file pharmaceutics-15-00192-s001.zip › pharmaceutics-2097968-supplementary.pdf]

Supplementary Material

Pharmaceutical Systems as a Strategy to Enhance the Stability of Oxytetracycline Hydrochloride Polymorphs in Solution

Maria S. Bueno [1,2], Marcela R. Longhi [1,2] and Claudia Garnero [1,2,*]

1 Departamento de Ciencias Farmacéuticas, Facultad de Ciencias Químicas, Universidad Nacional de Córdoba, Ciudad Universitaria, Haya de la Torre and Medina Allende, Science Building 2, Córdoba X5000HUA, Argentina
2 Unidad de Investigación y Desarrollo en Tecnología Farmacéutica, CONICET, Consejo Nacional de Investigaciones Científicas y Técnicas, UNITEFA, Córdoba X5000HUA, Argentina
* Correspondence: cgarnero@unc.edu.ar

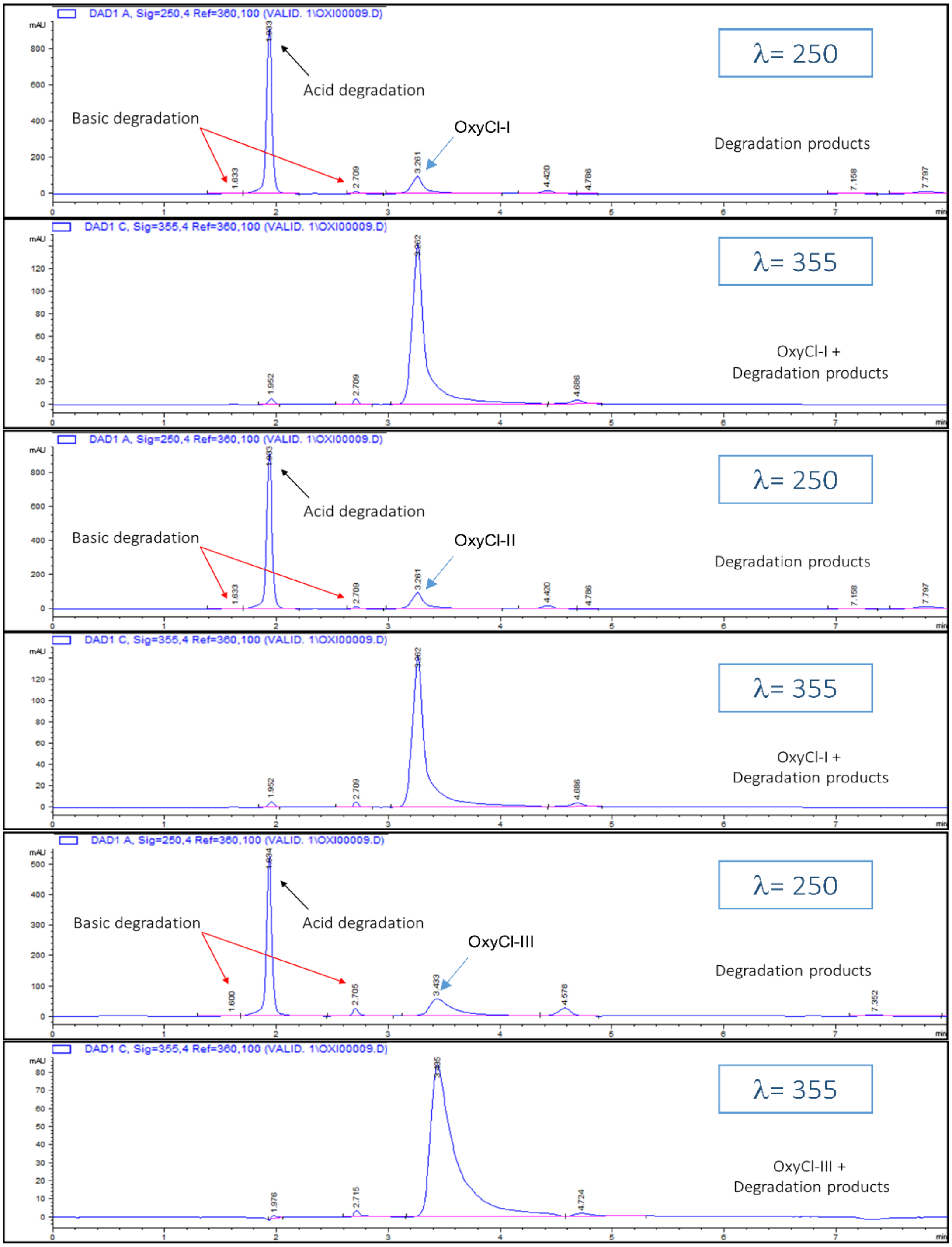

Figure S1. Chromatograms of OxyCl polymorphs and degradation products from acid and basic reactions.

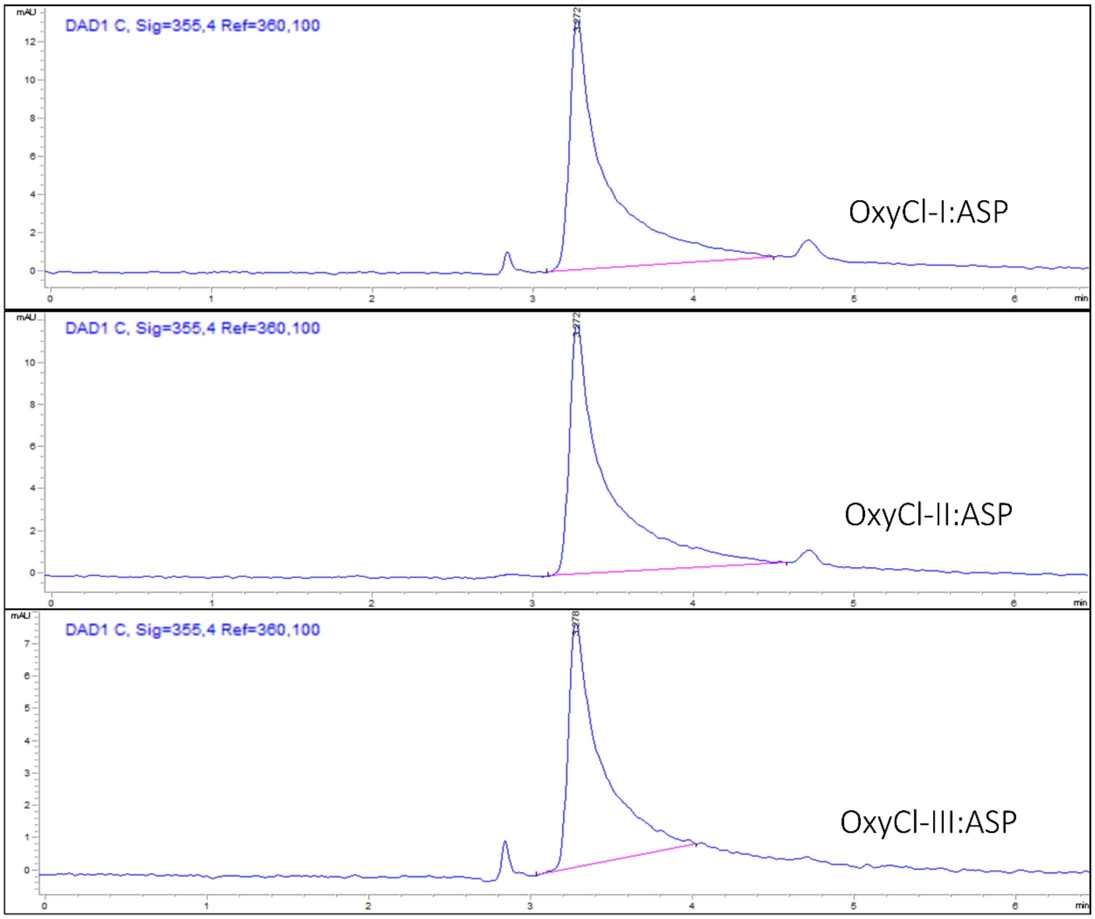

Figure S2. Chromatograms of binary systems with ASP in aqueous solution.

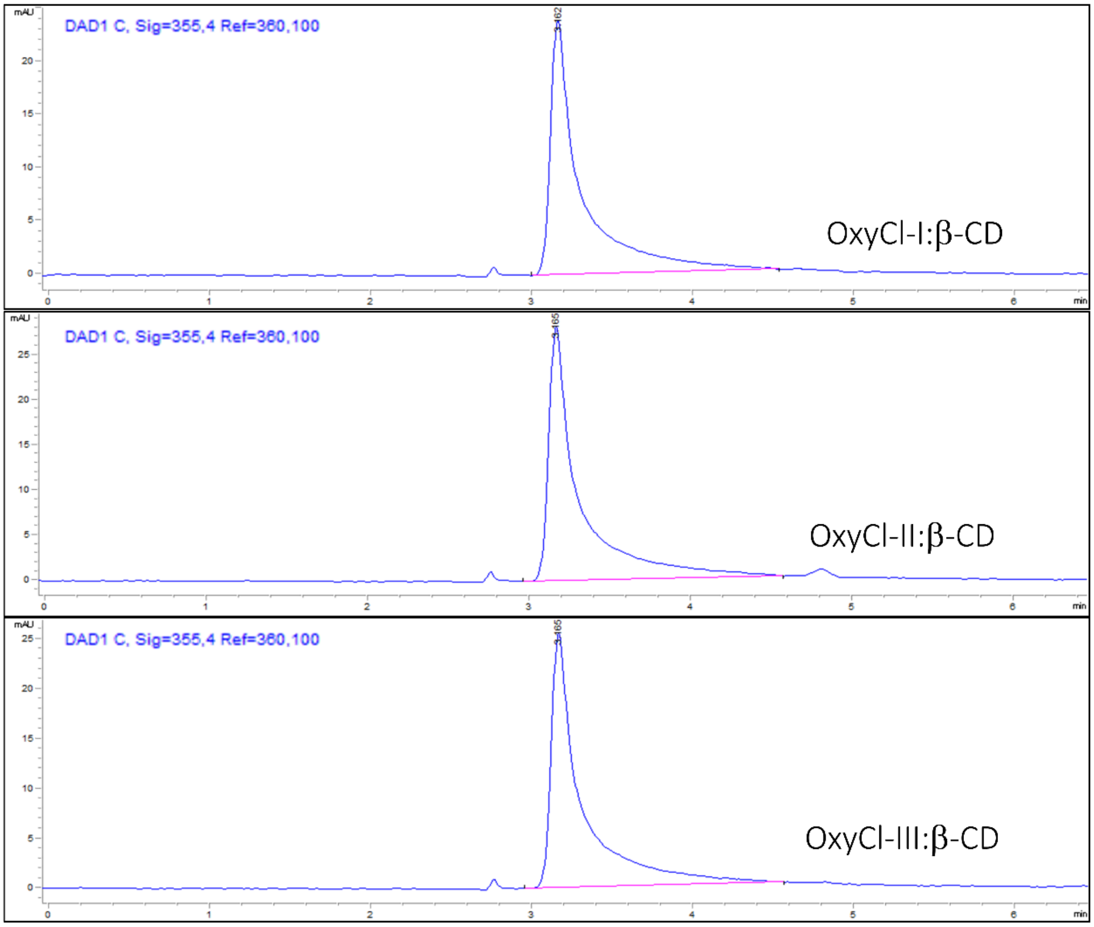

Figure S3. Chromatograms of binary systems with β-CD in aqueous solution.

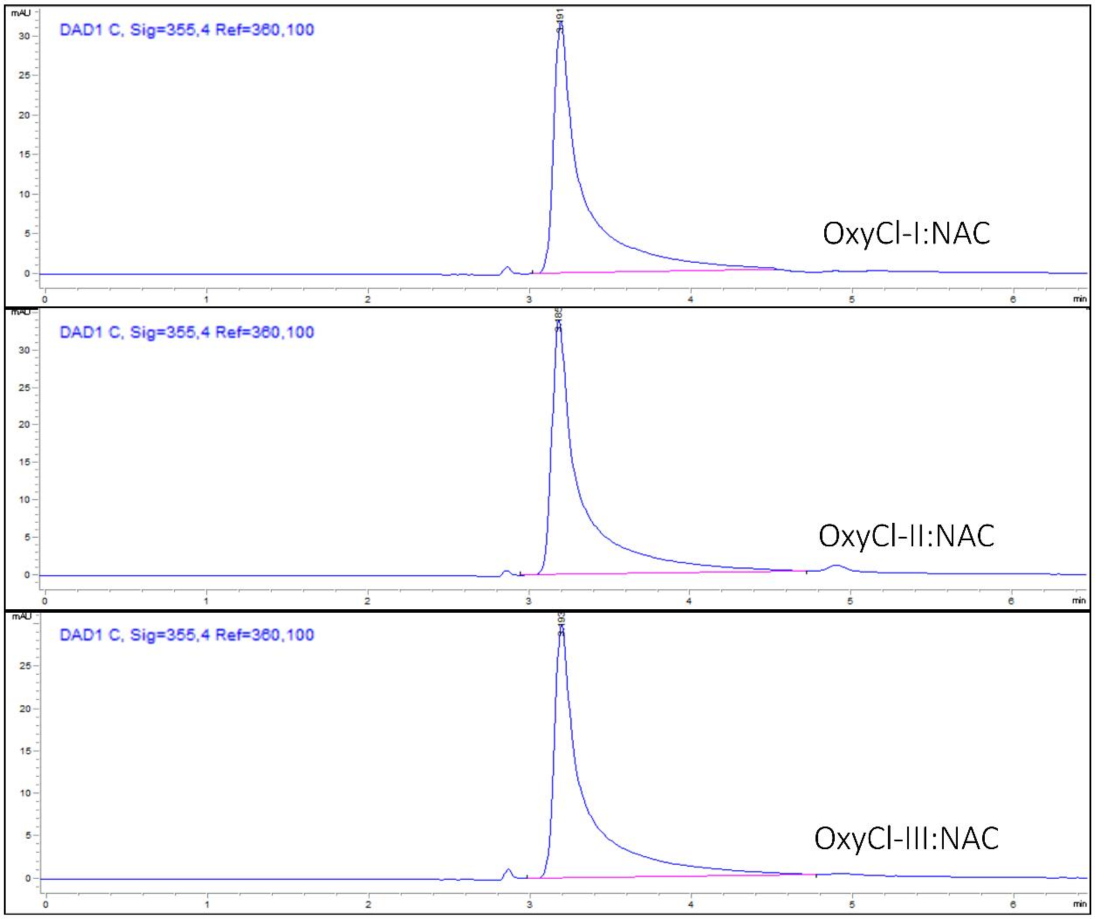

Figure S4. Chromatograms of binary systems with NAC in aqueous solution.

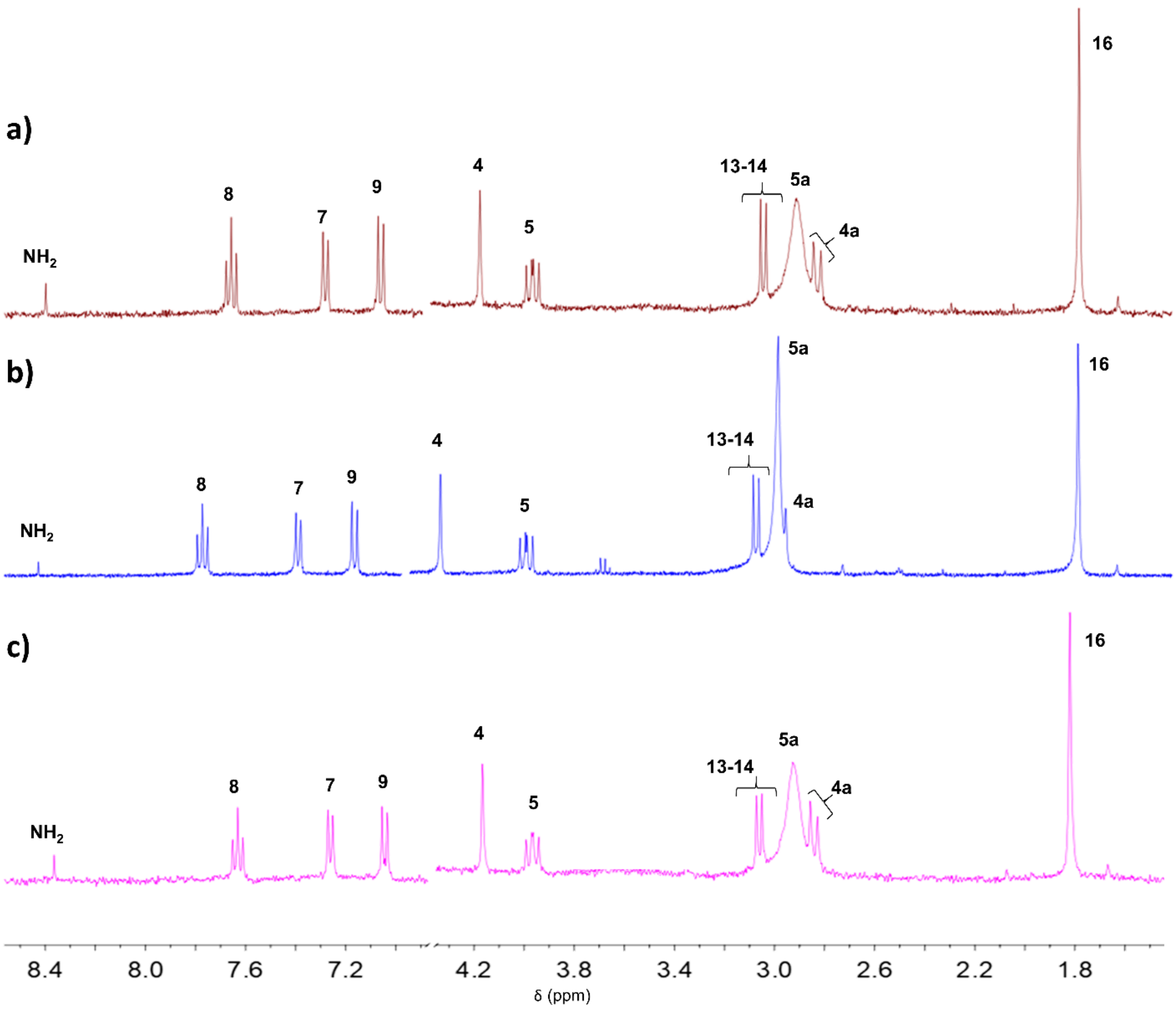

Figure S5. ^{1}H-NMR chemical assignment of a) OxyCl-I, b) OxyCl-II and c) OxyCl-III.
